# Influence of microbiology on endodontic failure. Literature review

**DOI:** 10.4317/medoral.22907

**Published:** 2019-05

**Authors:** Ilaria Prada, Pedro Micó-Muñoz, Teresa Giner-Lluesma, Pablo Micó-Martínez, Nicolás Collado-Castellano, Alberto Manzano-Saiz

**Affiliations:** 1 Licensed Dentist at Universidad Europea de Valencia, España; 2 Endodontic and dentistry Titular Professor, Universidad Europea de Valencia, España; 3 Endodontic and dentistry Associate Professor, Universidad Europea de Valencia, España; 4 Licensed Dentist at Universidad Europea de Valencia. Periodontology and Osteintegration Master at Universidad de Valencia, España

## Abstract

**Background:**

The main cause of endodontic failure is the persistence of microorganisms that cause an intraradicular or extratradicular infection and that become resistant to disinfection measures. The objective of this review is to identify the microbiota associated with endodontic failure, as well as the reasons why these microorganisms are capable of surviving basic disinfection measures.

**Material and Methods:**

Systematic search of scientific articles in the databases PubMed with the following keywords “Endodontic Infections”, “Endodontic Microbiology”, “Endodontic Failure”, “Enterococcus Faecalis”, “Endodontics Retreatment” was carried out. Case reports and articles with publication date prior to 2000 were not included in this review.

**Results:**

Most authors highlight *E. faecalis* as the main microorganism associated with endodontic failure, nevertheless there are recent studies that isolate, to a greater extent, other bacteria such as Fusobacterium nucleatum and Propionibacterium.

**Conclusions:**

These microorganisms have in common the following proprieties, which make them able to escape the disinfection measures: the ability to form a biofilm, to locate in areas unreachable to root canal instrumentation techniques, synergism, the ability to express survival genes and activate alternative metabolic pathways.

** Key words:**Endodontic infections, endodontic microbiologic, endodontic failure, enterococcus faecalis, endodontic retreatment.

## Introduction

Endodontic treatment is a reasonably predictable procedure with success rates of between 86% and 98%. The success or failure of this treatment is evaluated by the clinical signs and symptoms, as well as by the radiological findings of the treated tooth. The symptoms and clinical signs that define success are: the absence of pain, the disappearance of inflammation and fistulas, if they existed before treatment, as well as the maintenance of the functional and firm tooth in its alveolus. Radiographically, the complete healing of the existing periapical bone lesion and the normal appearance of the lamina dura for a period form 6 months to 24 months, will define success. Histologically, however, a complete repair of the periapical structures with absence of inflammatory cells must be produced (1).

The most frequent factors associated with endodontic treatment failure, due principally to the persistence of bacteria (intra and extra radicular), are deficient chemomechanical preparation and inadequate filling of the canal system. All this can occur as a result of improper preparations of the canals, fillings with lack of apical sealing, filtration in the restoration of the clinical crown, untreated canals, as well as iatrogenies such as apical transport, small access cavities, perforations, false pathways, instruments fractures etc, (1).

The main problem is that, in most cases, the apico-coronal seal is inadequate; therefore, tissue fluids rich in glycoproteins percolate into the root canal, providing a substrate to remaining microorganisms, which can proliferate and reach a sufficient number to generate or perpetuate a periradicular lesion (2). On the other hand, there are situations in which the sealed root canals can be contaminated from the oral cavity: filtrations through temporary or permanent restoration materials; fracture or loss of the restoration; fracture of the tooth structure; recurrent caries that expose the root filling material; or delay in the application of the definitive restoration material. In these circumstances, if the root filling doesn’t prevent the saliva percolation, the microorganisms can invade and re-colonize the canal system. Therefore, when a coronal exposure of the root filling occurs during a period of 30 days or more, it would be recommendable to do the endodontics again. In addition, given that temporary cements are water soluble and have a low compressive strength, the provisional coronal restoration should be replaced by the definitive one at the earliest opportunity (3).

Root canal bacteria can be isolated as planktonic cells, suspended in the liquid phase of the root canal and in the form of aggregates or congregatures adhered to root canals walls, giving place to several layers of biofilms. Biofilms are a model of bacterial growth where sessile cells interact to form dynamic communities linked to a solid substrate and located in a matrix of extracellular polymeric substances. The microorganisms that live in the same community must have the following characteristics: autopoiesis (having the ability to self-organize), homeostasis (resisting alterations of the environment in which they live), synergism (being more effective in groups than isolated) and the ability to respond to changes as a unit rather than as individuals (4).

To survive in a sealed duct, microorganisms have to endure the intracanal desinfection measures (chemomechanical preparation and intracanal drugs) and have to adapt to an environment with poor availability of nutrients. Therefore, only the few species that have these abilities may be involved in the endodontic treatment failure. In addition, bacteria located in areas such as apical deltas, isthmuses, lateral canals, irregularities and dentinal tubules, can often escape to endodontic disinfection procedures and it is probable that the bacteria nutrient supply remains unchanged after treatment. In contrast, the bacteria will not be able to survive if the substrate is drastically reduced or if the root filling does not allow the bacteria to access to perirradicular tissues. Nevertheless, resistant bacteria species will survive for relatively long periods by obtaining nutrients from tissue debris and dead cells. Furthermore, if the root filling does not provide an absolute seal, microfiltration of tissue fluids can provide a substrate for bacterial growth. The ability to survive in unfavorable conditions is very important for bacteria because they often experience periods of nutrient shortage. However, not always the microorganisms that manage to survive in these conditions are capable of causing endodontic failure. In fact, this will only occur if bacteria (their toxins and especially their endotoxins) are pathogenic, reach a sufficient number and access to periradicular tissues in order to induce or perpetuate periradicular lesions (2).

The objective of this literature review is to identify the main microorganisms that cause endodontic failure as well as the reasons that make them capable of surviving basic disinfection measures.

## Material and Methods

The article search was carried out by one researcher in the Pubmed database. *Endodontic, Infections, Microbiology, Enterococcus faecalis, Failure, Retreatment*, joined by the Boolean AND and limiting the search field of these words in the title and in the abstract were used as keywords.

The inclusion criteria for the articles selection were: Articles published after 2000, “full text” articles, journal articles with an “impact factor” greater than 1, literature review articles and research articles.

“Case report” articles and articles with publication date prior to 2000 were excluded.

## Results

**Figure 1:**
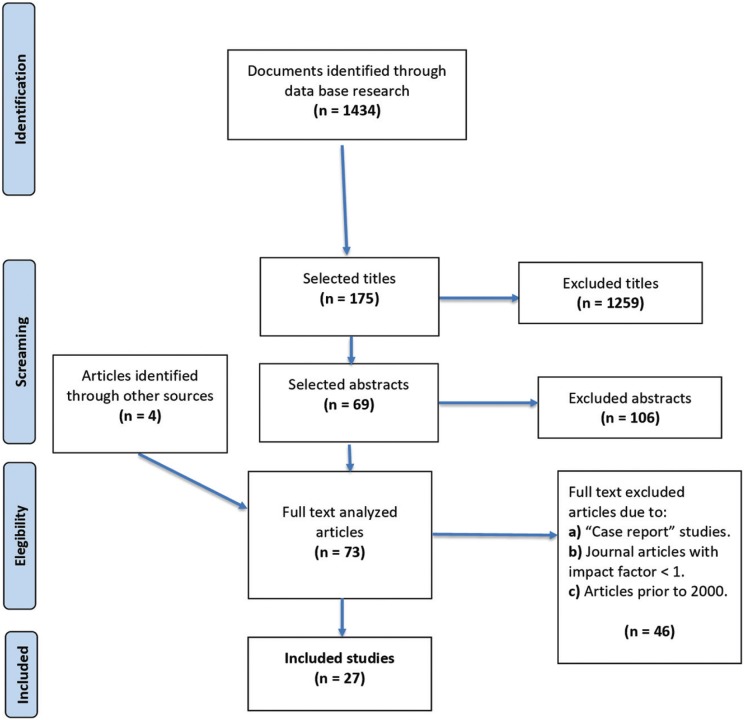
Flowchart.

A total of 1434 articles were initially found. Then, after reading the title of each article and taking into consideration the objectives of the work, 1259 articles were eliminated, reaching a total of 175 articles. After that, the summaries of the chosen articles were read and 106 were eliminated because they were not considered relevant for the review. Finally there were 69 articles for the full text review, which 46 articles were excluded for not meeting the inclusion criteria. In addition to these articles, another 4 articles were found after reading the references of the initially included articles. The articles taken into consideration were finally 27 (Fig. 1).

14 out of 27 articles were “*in vivo*” studies (5-18), which objective was to investigate the microorganisms that could be isolated in teeth with endodontic failure and apical periodontitis (AP) or during the re-treatment process. 2 out of 27 articles were “*ex vivo*” studies (19), (20) which objectives were to investigate the microorganisms isolated in the apical portion of teeth with apical periodontitis (20) and to evaluate the presence of intrarradicular or extrarradicular biofilm in teeth with or without AP (19). 3 out of 27 articles were “*in vitro*” studies (21-23) that analyzed the Enterococcus faecalis resistance mechanisms and the stress response. 8 out 27 articles were “review” (4,24-30); specifically, 4 described all the microorganisms related with endodontic failure and enumerate the characteristics of the most frequently isolated bacteria that make them resistant to the disinfection measures (4,26,29,30) focused on Enterococcus faecalis and its characteristics (24,25,27) and 1 focused on Treponema species (28).

## Discussion

The main cause of endodontic infections, and therefore also of endodontic treatment failure, is, as already sated, the presence of microorganisms isolated as planktonic cells or biofilms. Biofilm provide pathogens a more favorable habitat to live in and a more efficient metabolic diversity. In addiction, these coordinated functional communities offer bacteria protection against other competitive microorganisms, antimicrobial agents and host defenses, increasing therefore its pathogenicity (19). Nevertheless, it is difficult to determine if an infection is caused by a biofilm. Due to this, Parsek and Singh (31), in 2003, proposed some criteria to define the infections caused by biofilms. Bacteria have to be attached or associated to a surface, the examination of the infected tissues has to show some microcolonies surrounded by extracellular matrix, the infection must be limited to a specific location and must be difficult or impossible to eradicate with antibiotics. The location of this biofilm can be both intra-radicular and extra-radicular. Most of the time, in 77% of cases, this biofilm is usually intra-radicular, while only 6% represents the extra-radicular portion. Furthermore, it has been discovered that, unlike what was expected, the presence of intra-radicular biofilm is usually associated with periapical lesions of long evolution, in particular, it is found with a statistically higher frequency in cysts than in granulomas (19).

Different studies have analyzed root canal biofilms composition of teeth with apical periodontitis after endodontic treatment, with heterogeneous results regarding the mostly present pathogen in unsuccessful cases. The most important outcomes founded in the different studies are summarized in  Table 1, Table 1 continue,  Table 2.

**Table 1:**
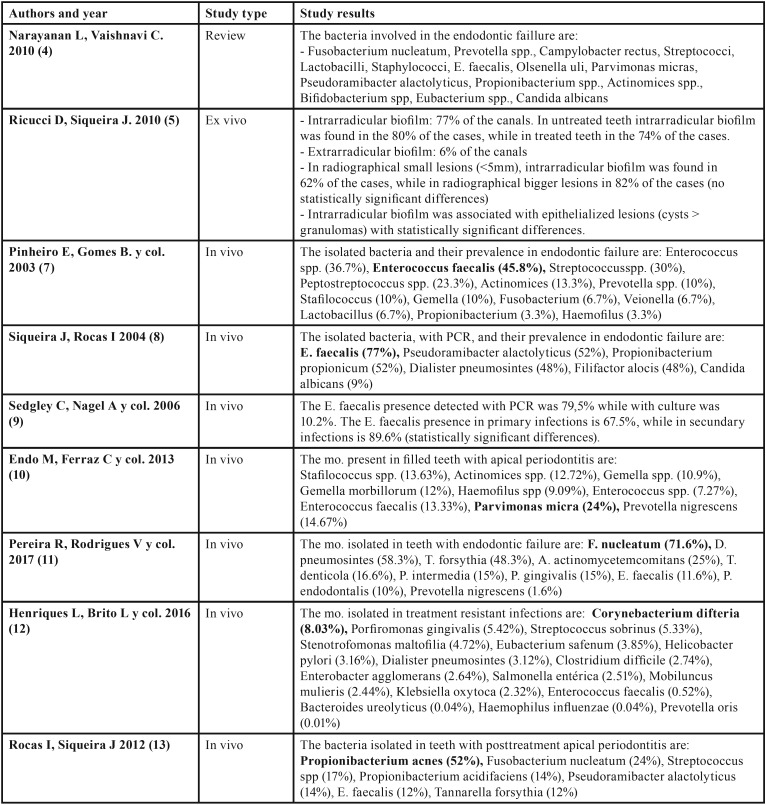
Discussion tables with main outcomes of each article classified by study type.

**Table 1:**
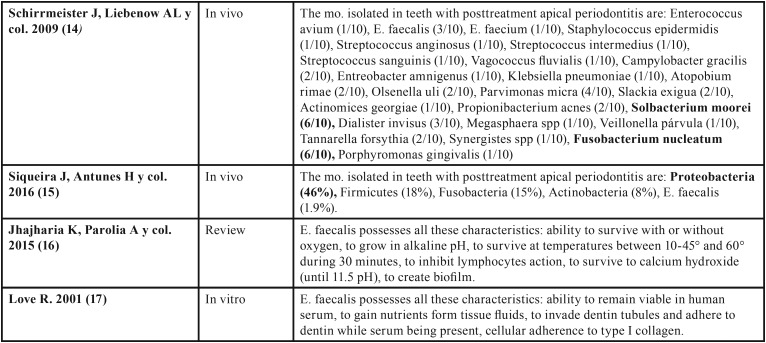
Discussion tables with main outcomes of each article classified by study type.

**Table 2:**
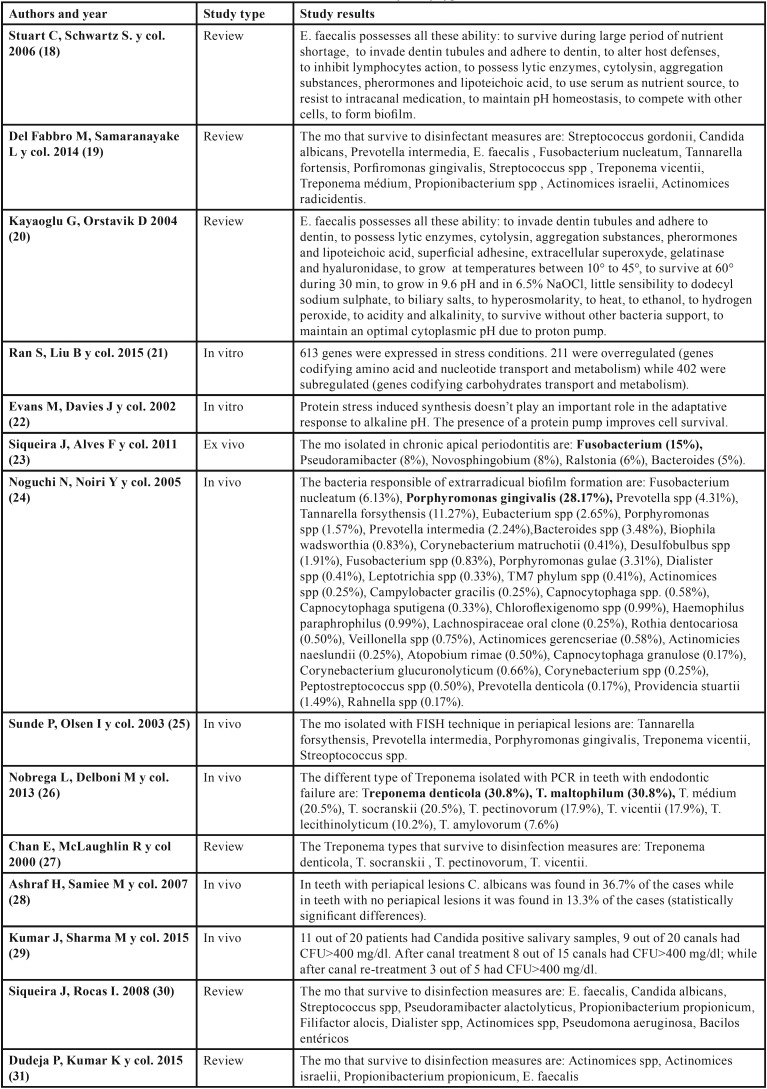
Discussion tables with main outcomes of each article classified by study type.

Pinheiro *et al.* (5), in 2003, highlighted that *E. faecalis* is, statistically, the most prevalent microorganism found (45.8%) in root canals previously filled, followed by *Fusobacterium*, (6.7%) and *Propionibacterium* (3.3%). Similar results were reported by Siquiera and Roças (6) and Sedgley *et al.* (7), using the Polymerase Chain Reaction (PCR) technique. They observed respectively, a 77% and 79.5% prevalence of *E. faecalis*. These authors also found that the presence of *E. faecalis* is more frequent in secondary infections (89.6%) than in primary infections (67.5%), with statistically significant differences (7). However, in other studies, *E. facealis* does not stand out as the main responsible for endodontic failure. Even so, it is almost always present but in smaller percentages: 13.33% (8), 11.6% (9), 0.52% (10), 12% (11), 30% (12), and 1.9% (13). This is why, many studies have been carried out to identify mechanisms that make this microorganism so resistant to the disinfection measures applied in the endodontic treatment. *E. faecalis* is a gram-positive, anaerobic facultative coco classifiable as an opportunistic pathogen. It has different mechanisms that allow it to survive in an unfavorable environment. For example, the ability to grow with or without oxygen, to grow at an alkaline pH, to survive at temperatures between 10° - 60° to suppress the lymphocytes action, to survive Ca(OH)2 solutions with pH 11.5, and ability to form a biofilm (24). To these survival mechanisms, we can also add the ability of *E. faecalis* to live without nutrients, to survive in the presence of intracanal drugs and irrigants, to survive at high salinity, to acquire antibiotic resistance (4), in particular to erythromycin and azithromycin (5), to invade dentinal tubules, to use fluids from the periodontal ligament (LPO) as nutrients and to adhere to collagen (4). The role of collagen varies depending on whether it is immobilized type I collagen or free collagen: the former increases the ability of *E. faecalis* to adhere to dentin, while the latter inhibits its adhesion capacity with statistically significant differences (21). In addition to the characteristics enumerated so far, the study by Stuart *et al.* (25), in 2006, adds the capacity of *E. faecalis* to alter host responses, to possess lytic enzymes, to maintain pH homeostasis, to compete with other cells and to use serum as a nutrients source. Human serum, therefore, guarantees the viability of *E. faecalis*, allowing its adhesion to dentin and invading the dentinal tubules (21). Moreover, this bacteria has the capability to survive to NaOCl concentrations of up to 6.5%, to acquire and share extrachromosomal elements, to encode virulence factors, to produce pathological changes by secreting endotoxins that provoke inflammatory responses (26), to induce hydroxyapatite reprecipitation in mature biofilms, to form a calcified biofilm and adhere to dentin. Additionaly, this microorganism has lower sensitivity to lethal levels of sodium dodecylsulfate, hyperosmolarity, heat, ethanol, hydrogen peroxide, acidity and alkalinity. Furthermore, *E. faecalis* is able to survive without the support of other bacteria, to possess aggregation substances and surface adesines, lipoteichoic acid, extracellular superoxide, gelatinase, hyaluronidase and cytolysin (27). The aggregation substances are bacterial adhesive substances encoded by plasmids, which mediate the contact between the donor and recipient bacteria, facilitating the exchange of the plasmid. They act by increasing the adhesion during the bacterial conjugation process and also favor the adhesion of *E. faecalis* to numerous eukaryotic cells; they also enforce the bacteria binding strength to type I collagen and the resistance to the neutrophils action, which makes it a protective factor against host defenses. Microorganisms, such as *E. faecalis*, that possess these aggregation substances are capable of inducing the proliferation of T cells, with the consequent release of β tumor necrosis factors (TNF-β) and γ interferon (INF-γ), and to activate the macrophages to release α tumor necrosis factors (TNF-α). TNF are involved in bone resorption, while INF-γ will increase the production of hydrogen peroxide and superoxide anions that cause cellular and tissue damage. Surface adhesins give *E. faecalis* the ability to adhere to different substances, such as abiotic surfaces (necessary for biofilm formation), other bacteria (permitting nutrients and genes exchange), to collagen, serum and dentin (27).

Lipoteichoic acid is an amphipathic molecule composed of a polyglycerol phosphate chain. Its release can cause apoptosis in several cells, like osteoblasts, osteoclasts, LPO fibroblasts, macrophages and neutrophils. They can also stimulate leukocytes to release several inflammation mediators, including TNF-α, interleukin 1 beta (IL-1β), interleukin 6 (IL-6) and interleukin 8 (IL-8). Superoxide anion is a highly reactive oxygen radical that is involved in tissue and cell damage, producing bone loss in cases of chronic apical periodontitis. Gelatinase is an extracellular metal-proteinase that contains zinc and can hydrolyze gelatin and collagen, thus causing periapical inflammation. Hyaluronidase acts on hyaluronic acid and is a degrading enzyme associated with tissue damage. This enzyme depolymerizes the mucopolysaccharides of the connective tissue thereby increasing bacterial invasion, and also contributes to the obtaining of nutrients for the bacterium since it is capable of degrading the disaccharides transported to the interior of the cell to be metabolized. Another activity that can be achieved by hyaluronidase is bacterial degradation, favoring the prevalence of those bacteria that possess this type of enzyme, which also allows them to migrate from the root canals to the periapical tissues. Cytolysin, finally, is a toxin encoded by plasmids capable of exerting a lytic action against a broad spectrum of Gram-positive and Gram-negative bacteria, thus favoring the survival of those microorganisms that possess it (27). Beside all these factors and characteristics that guarantee *E. faecalis* survival, Ran *et al.* (22) observed that, exposing these bacteria to stress conditions (pH10) it expresses 613 specific genes, of which 211 are upregulated and 402 downregulated. The overregulated genes corresponded mainly to those encoding aminoacids and nucleotides transport and metabolism. This indicates that, in stress condition, *E. faecalis* is able to use certain aminoacids as energy and carbon, to promote pyrimidine biosynthesis, leading to bacteria virulence increase. The downregulated genes, on the other hand, corresponded to genes involved in carbohydrates transport and metabolism (22). Furthermore, it has been demostrated that *E. faecalis*, under these conditions, prefers to use other metabolic pathways, rather than adenine triphosphate (ATP) synthetase, such as phosphoenolpyruvate (PEP) that transports sugars such as glucose and fructose into the bacteria. In addition to the regulation of genes, it has been shown that *E. faecalis* is able to synthesize proteins to cope with stress conditions, but its production does not guarantee its survival at a very high pH (23). The presence of a functioning proton pomp inhibitor (CCCP) is the most important mechanism that allows the bacteria to regulate the pH. When the pH, due to the application of Ca(OH)2, becomes very alkaline, this pump is activated and allows protons to be transported to the interior of the cell to acidify the cytoplasm and, therefore, allows the cell to survive. However, this pump will work until a limit level of pH of 11.5 is reached, at this value the pump becomes saturated and stops functioning, thus leading to cell death (23).

Recent studies do not highlight the bacterium *E. faecalis* as the main responsible for endodontic failure. Thus, Endo *et al.* (8), in 2013, found that the most frequently isolated microorganism, in 24% of cases, was *Parvimonas micra*. Schirrmeister *et al.* (12) also detected this bacteria in his study, however, unlike the previous investigation, *Parvimonas micra* was the third most prevalent bacteria, follwed by Solbacterium moorei and *Fusobacterium nucleatum*. Pereira and col (9), in 2017, highlighted the *Fusobacterium nucleatum*, as the most prevalent bacteria (71.6%) in teeth with post-treatment periodontitis. A similar study, conducted by Siqueira *et al.* (20) also pointed up this bacteria as the most prevalent (15%). Rôças and Siqueira (11) again underlined the importance of *Fusobacterium nucleatum* in 2012, when it was isolated with a 24% prevalence, as the second most frequent bacterium. Henriques *et al.* (10), however, in 2016, pointed out *Corinebacterium diphtheria* as the most important bacteria related to endodontic failure. However, Siqueira *et al.* (13), in 2016, discovered that in the apical portion of the root canals with post-treatment AP it is more frequent to find, in 46% of cases, the proteobacteria. A few years before, in 2012, the same author, together with Rôças (11), isolated *Propionibacterium* acnes (52% prevalence), as the most frequently present microorganism in cases of post-treatment apical periodontitis. The same authors, in another study (6) identified *Propionibacterium* propionicum as the second most prevalent bacteria (52% prevalence) after *E. faecalis*. Other authors who confirmed the presence of *Propionibacterium* acnes in teeth with endodontic failure were Schirrmeister *et al.*, who detected this bacterium in 2 cases out of 10 (12). What makes this bacterium resistant to disinfection measures is its ability to search for alternative sources of nutrients, to escape from host’s defenses, to survive in granulation tissue present outside the canals and its ability to adhere, coaggregate and survive in extra-radicular areas (26). Another microorganism capable of surviving in the extra-radicular area is Porphyromonas gingivalis, which, with 28.17%, was classified, in the study by Noguchi *et al.* (14) in 2005, as the microorganism most frequently observed in the areas that are in close contact with the root surface, being probably a pioneer bacterium in the colonization of the extra-radicular area. The presence of Porphyromonas gingivalis has also been detected in the Schirrmeister’s study (12), but with a lower prevalence, 1 case out of 10. Sunde *et al.* (15) studied the survival mechanisms that this bacteria uses both in periodontitis and periapical lesions: synergic interaction to share nutrients, virulence factors and protection mechanisms against host defenses. In addition to *Porphyromonas*, the bacteria *Tannarella forsythensis, Prevotella intermedia, Streptococcus spp* and *Treponema vicentii* were also highlighted (15). Another author who underlines the importance of Treponema in cases requiring endodontic re-treatment is Nobrega *et al.* (16), in 2013. However, they stand out two other species: *Treponema denticola* and *Treponema maltophilum* (30.8% prevalence) in comparison to *Treponema vicentii* (17.9%). Treponema species are resistant to endodontic treatment because they are able to produce proteolytic enzymes, adhere to and invade host cells, penetrate tissues due to its great mobility, inhibit neutrophils and polymorphonuclear leukocytes’ function, as well as possess lipopolysaccharides (LPS) and release endotoxins that exacerbate inflammatory response and tissue damage (28). Another microorganism that has always been associated with endodontic failure is Candida albicans. This fungus is present, to a greater degree, in teeth with periapical lesions (36.7%) than in teeth without these lesions (13.3%) (17). Siqueira and Rôças (6) also established that *C. albicans* is the most prevalent fungus found in previously sealed root canals. In contrast, the study conducted by Kumar *et al.* (18), in 2015, which analyzed the presence of *C. albicans* in those teeth that needed endodontic retreatment, observed that after primary treatment, 8 out of 15 canals, 53.3%, had a number of colony forming units (CFU) > 400 mg/dl, while after reendodontics, 3 out of 5 canals had a CFU > 400 mg/dl. These data indicate that, despite all the disinfection methods applied, both mechanical and chemical, the canals were still contaminated by Candida, thus was able to avoid these measures (18).

**Figure 2:**
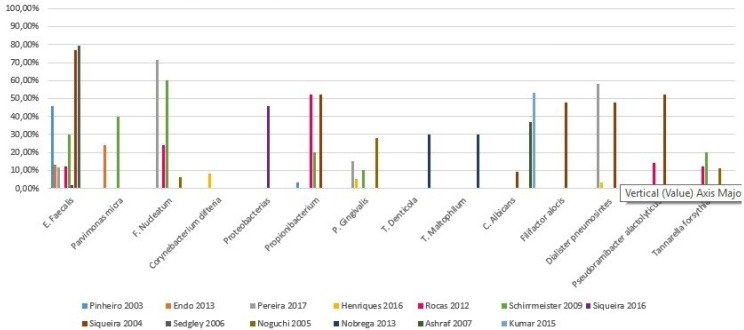
Microorganisms prevalence in endodontic failure.

Many other bacteria have also been identified with high prevalences 48%-60%: *Filiphactor alocis* 48%, *Dialister pneumosintes* 48% - 58.3%, *Pseudoramibacter alactolyticus* 52% and *Tannarella forsythia* 48.3% (6) (9) (Fig. 2). There are other microorganisms that can be isolated less frequently in the root canals of those teeth that present endodontic treatment failure: *Pseudoramibacter, Novosphingobium, Ralstonia, Bacteroides, Firmicutes, Actinobacteria, Enterococcus avium, E. faecium, Staphylococcus epidermis, Streptococcus anguinosus, Streptococcus intermedius, Streptococcus sanguinis, Vagococcus fluvialis, Campylobacter gracilis, Enterobacter amnigenus, Klebsiella pneumoniae, Atopobium rimae, Oslenella uli, Slackia exigua, Actinomices georgiae, Dialister invisus, Megasphera spp, Veillonella párvula, Tannarella forsythia, Synergistes spp, , Propionibacterium acidifaciens, Streptococcus spp, Rahnella spp, Providencia stuartii, Prevotella denticola, Peptostreptococcus spp, Corynebacyerium spp, Corynebacterium glucuronolyticum, Capnocitophaga granulosae, Actinomicies naselundii, Actinomicies gerencseriae, Veillonella spp, Rothia dentocariosa, Lachnospiraceae oral clone, Haemophilus paraphrophilus, Chloroflexigenomo spp, Capnocytophaga spp, Capnocytophaga sputigena, Actinomicies spp, TM7 phylum spp, Leptotrichia spp, Dialister spp, Porphyromonas gulae, Desulfobulbus spp, Corynebacterium martuchotii, Biophila wadsworthia, Porphyromonas spp, Eubacterium spp, Prevotella oris, Streptococcus sobrinus, Stenotrofomonas maltofilia, Eubacterium safenum, Helicobacter pylori, Clostridium difficile, Enterobacter agglomerans, Salmonella entérica, Mobiluncus mulieris, Klebsiella oxytoca, Bacteroides ureolyticus, Haemophilus influenzae, Agregatibacter actinomycetemcomitans, Porphiromonas endodontalis, Prevotella nigescens, Gemella spp, Gemella morbillorum, Campylobacter rectus, Lactobacillus spp, Bifidobacterium spp, Actinomices israelii, Pseudomona aeruginosa, Bacilos entéricos, Streptococcus gordonii*, (4-6,8-14,20,26,29,30).

These microorganisms have specific characteristics that enable them to avoid mechanical and chemical instrumentation carried out during endodontic treatment. These can be summarized as follows: capacity to create strongly attached biofilms colonize distant areas from the main canals (apical deltas, isthmuses, lateral canals) that are almost impossible to reach with the instrumentation, being protected by tissue residues, dentin, serum and dead cells that inactivate or diminish the efficiency of antimicrobial agents. Furthermore, these bacteria must be intrinsically resistant to antimicrobial agents, be able to adapt by activating survival genes and using alternative metabolic pathways, must possess bacterial aggregation capacity and synergism, as well as must be located in areas where nutrient sources are minimally affected (29).
